# The Gas2 family protein Pigs is a microtubule +TIP that affects cytoskeleton organisation

**DOI:** 10.1242/jcs.176230

**Published:** 2016-01-01

**Authors:** Gemma C. Girdler, Derek A. Applewhite, Wick M. G. Perry, Stephen L. Rogers, Katja Röper

**Affiliations:** 1 MRC-Laboratory of Molecular Biology, Cambridge Biomedical Campus, Francis Crick Avenue, Cambridge CB2 0QH, UK; 2 Department of Biology &Carolina Center for Genome Sciences, The University of North Carolina at Chapel Hill, Campus Box 3280, 422 Fordham Hall, Chapel Hill, NC 27599-3280, USA; 3 Department of Biology, Reed College, 3203 SE Woodstock Boulevard, Portland, OR 97202, USA

**Keywords:** Cytoskeleton, Cytolinker, Actin, Microtubule, Gas2-like, *Drosophila*

## Abstract

Coordination between different cytoskeletal systems is crucial for many cell biological functions, including cell migration and mitosis, and also plays an important role during tissue morphogenesis. Proteins of the class of cytoskeletal crosslinkers, or cytolinkers, have the ability to interact with more than one cytoskeletal system at a time and are prime candidates to mediate any coordination. One such class comprises the Gas2-like proteins, combining a conserved calponin-homology-type actin-binding domain and a Gas2 domain predicted to bind microtubules (MTs). This domain combination is also found in spectraplakins, huge cytolinkers that play important roles in many tissues in both invertebrates and vertebrates. Here, we dissect the ability of the single *Drosophila* Gas2-like protein Pigs to interact with both actin and MT cytoskeletons, both *in vitro* and *in vivo*, and illustrate complex regulatory interactions that determine the localisation of Pigs to and its effects on the cytoskeleton.

## Abstract (precis)

**Summary:** Detailed analysis of the ability of the *Drosophila* cytolinker protein Pigs to modulate its cytoskeleton-interacting functions (+TIP tracking, microtubule shaft binding, actin binding and actin–microtubule crosslinking).

## INTRODUCTION

The actin and microtubule (MT) cytoskeletons are important for many cell biological functions (Rodriguez et al., 2003). Both cytoskeletal networks have often been studied independently, and research has identified many factors that modulate the dynamic behaviour and structure of each system. However, many processes require that the behaviour of the actin and MT cytoskeletons is closely coordinated. This is very apparent during, for instance, cell division, where spindle MTs determine the site of assembly of the contractile actomyosin ring (Kunda and Baum, 2009), or during growth cone steering, where axonal MTs affect growth cone actin dynamics (Geraldo and Gordon-Weeks, 2009). Close coordination between actin and MTs is also required to direct the cell shape changes that drive tissue morphogenesis. Even though many accessory cytoskeletal proteins that remodel actin or MTs have been identified, only a few classes of proteins have been shown to be able to bind to both simultaneously and thereby crosslink and coordinate the two networks. These proteins include some MT plus-end binding proteins, such as CLASPS (Tsvetkov et al., 2007), the tumour suppressor Adenomatous polyposis coli (APC) (Aoki and Taketo, 2007), mDia (Bartolini et al., 2012) and the large multidomain plakin and spectraplakin proteins (Röper et al., 2002; Brown, 2008; Suozzi et al., 2012). Several proteins, including other MT-plus-end-binding proteins, such as EB1 (also known as MAPRE1 in mammals), and CLIP170 (also known as CLIP1 in mammals, and as CLIP190 in *Drosophila*) can bind to MTs and interact indirectly with actin (Fukata et al., 2002; Wen et al., 2004). All of these proteins have been conserved throughout evolution, and consistent with this, have been shown to have essential roles in a variety of cellular processes, including axon growth (Sanchez-Soriano et al., 2009; Alves-Silva et al., 2012), cell migration (Kodama et al., 2003; Drabek et al., 2006) and wound healing (Wu et al., 2008).

Spectraplakins are huge proteins with many interaction domains that allow binding to all cytoskeletal systems (Sun et al., 2001; Röper et al., 2002). Vertebrates have two spectraplakins, MACF1 and BPAG1, whereas *Drosophila* has only one, the protein Short Stop (Shot) (Bernier et al., 1996; Gregory and Brown, 1998; Lee et al., 2000; Gong et al., 2001; Röper et al., 2002; Suozzi et al., 2012). Shot is important for many processes during development, where it plays roles during axon pathfinding (Lee and Kolodziej, 2002b), maintenance of epithelial integrity (Röper and Brown, 2003), integrin adhesion (Gregory and Brown, 1998), oocyte determination (Röper and Brown, 2004), tracheal anastomosis (Lee and Kolodziej, 2002a) and tubulogenesis (Booth et al., 2014). In all cases, the ability of Shot to influence the cytoskeleton is key to its role, and in some cases it has been clearly shown that the crosslinking ability is required for function (Lee and Kolodziej, 2002b; Sanchez-Soriano et al., 2009). The domains of Shot that mediate its interaction with the cytoskeleton are two N-terminal calponin-homology (CH)-type actin-binding domains, and a C-terminal Gas2 domain, in combination with surrounding sequences, as well as Sx(I/L)P motifs at the very C-terminus (Lee et al., 2000; Wu et al., 2008; Applewhite et al., 2010). CH domains come in a variety of flavours. Actin-binding is usually mediated by two paired domains, a type 1 and a type 2 CH domain (Sjoblom et al., 2008), and this is also the case in Shot. The type 1 domain, in isolation, will bind actin, whereas the type 2 domain does not. Further subfamilies of CH domains are also involved in mediating protein–protein interactions rather than actin binding, and some can even mediate interaction with MTs rather than actin (Gimona et al., 2002). The MT-binding Gas2 domain was originally identified in the protein Gas2 (Brancolini et al., 1992). Analysis of this domain in isolation compared to in a larger protein context suggests that MT binding is mediated by the Gas2 domain in combination with surrounding sequences (Sun et al., 2001; Goriounov et al., 2003; Sanchez-Soriano et al., 2009).

Apart from the Spectraplakins, the only other known family of proteins that also contains a single CH domain paired with a Gas2 domain is the Gas2-like family of proteins. In vertebrates it consists of four members, Gas2 and Gas2-like (Gas2l)1–3 (Brancolini et al., 1992; Goriounov et al., 2003; Stroud et al., 2011). Structure function analysis of Gas2l1 and Gas2l3 in heterologous expression systems has shown that these proteins can indeed bind to actin and MTs (Stroud et al., 2011; Wolter et al., 2012). Proposed functions for the different Gas2-like family members have only recently emerged and include a role for Gas2l3 in the cell cycle as a target of the DREAM complex (Wolter et al., 2012) and a potential role in cell abscission after division (Pe'er et al., 2013). *Drosophila* has only one Gas2-like family member called Pigs, with a proposed function as a cytolinker whose activity is regulated by Notch signalling (Pines et al., 2010). With single CH domains being able to confer a wealth of interactions, not only to actin but possibly even to MTs, and with Gas2 domains being able to mediate MT binding, but only in the context of surrounding sequences, we wanted to dissect the function of Pigs further and determine in which ways it could interact and influence the cytoskeleton.

To this end, we carried out a detailed structure–function analysis of Pigs both *in vitro* in *Drosophila* tissue culture cells and *in vivo* in *Drosophila* tissues. Pigs bound both actin and MTs, but was also an efficient MT plus-end tracker, and our analysis suggests a complex regulation of its ability to interact and crosslink actin and MTs.

## RESULTS

### Pigs is an MT plus-end-tracking protein in cultured cells and in fly tissues

**Fig. 1.**
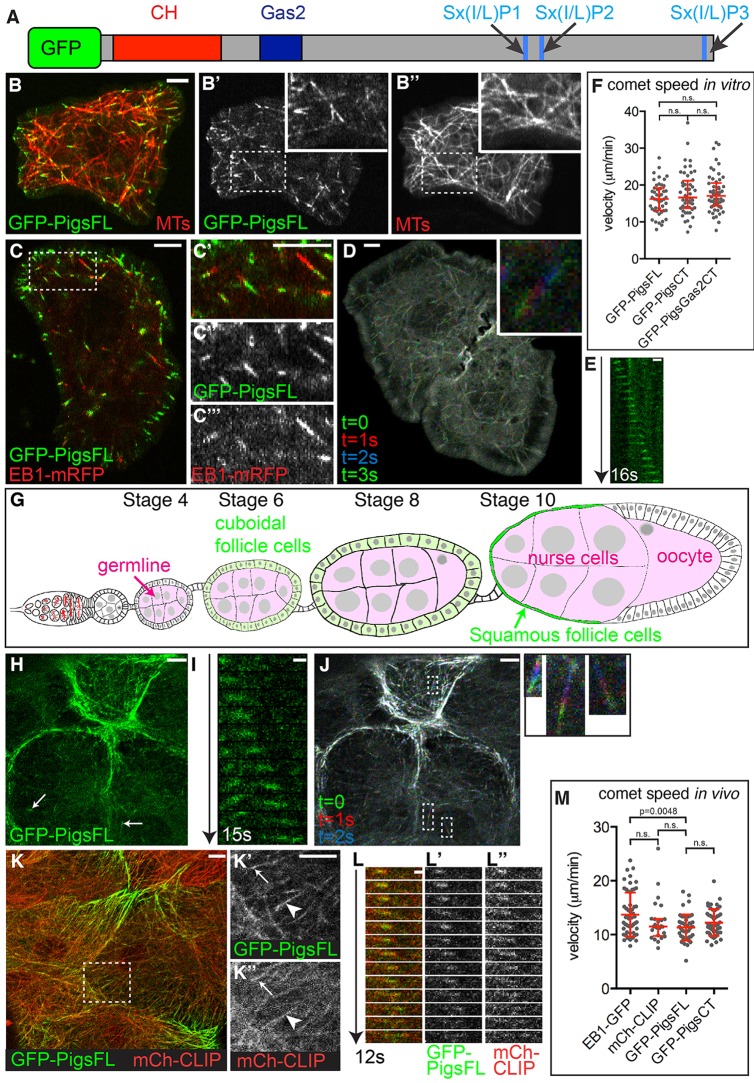
**Pigs is an** **MT +TIP in *Drosophila* tissue culture cells and *Drosophila* tissues *in vivo*.** (A) Schematic of the Pigs protein. CH, Calponin homology domain; Gas2, Gas2 domain; Sx(I/L)P1– Sx(I/L)P3, predicted plus-end MT localisation motifs. (B) GFP–PigsFL (green, B′) localises to the ends of MTs labelled with mCherry–tubulin (red, B″) in S2 cells. Insets show a magnification of the boxed region. (C) GFP–PigsFL (green, C″) partially colocalises with EB1–mRFP (red, C‴) at MT plus-ends in S2 cells. C′–C‴ show a magnification of the boxed region. (D) Frames from a time-lapse movie of a GFP–PigsFL-expressing S2R+ cell shown as a maximum intensity projection of four consecutive frames, pseudocoloured in green (0 s), red (+1 s), blue (+2 s), and then green (+3 s) again. The inset shows a magnified single comet. See Movie 1. (E) Kymograph of GFP–PigsFL tracking an MT plus-end in a S2R+ cell over 16 s. (F) Analysis of comet speeds in S2R+ cells expressing GFP–PigsFL, GFP–PigsCT or GFP–PigsGas2CT. Shown are all data points, the median and the interquartile range. n.s., not significant (Mann–Whitney test). (G) Schematic of *Drosophila* oogenesis. Pigs was expressed either in squamous follicle cells (green) or the nurse cells of the germline (pink). (H) Still picture from a time-lapse movie of GFP–PigsFL expressed in the squamous follicle cells of a *Drosophila* ovary in culture at stage 10. Arrows point to plus-end comets marked by GFP–PigsFL. See Movie 2. (I) Kymograph of GFP–Pigs tracking an MT plus-end in the ovary over 15 s. (J) Maximum intensity projection of pseudocoloured frames from a time-lapse movie of ovaries expressing GFP–PigsFL. Green (0 s), red (+1 s), blue (+2 s). Boxes indicate single comets that are shown magnified to the right. (K) Still from a time-lapse movie of *Drosophila* ovary squamous follicle cells at stage 10 expressing GFP–PigsFL (green, K′) at the end of MTs labelled by mCherry–CLIP170 (mCh-CLIP; red, K″). Arrow points to a comet, arrowhead points to Pigs colocalisation with lattice MTs. K′–K″ show a magnification of the boxed region. (L) Kymograph of GFP–PigsFL (L′, green) and mCherry–CLIP170 (L″, red) tracking an MT plus-end in the ovary squamous follicle cells over 12 s. (M) Analysis of comet speeds *in vivo* in squamous follicle cells expressing EB1–GFP, mCh-CLIP, GFP–PigsFL or GFP–PigsCT. Shown are all data points, the median and the interquartile range. Significance was tested using Mann–Whitney test; n.s, not significant. Scale bars: 5 µm (B–D, H, J, K); 1 µm (E, I , L).

To assess the localisation of Pigs, we expressed GFP-tagged full length Pigs (GFP–PigsFL, Fig. 1A) using copper inducible vectors (pMT) in *Drosophila* cells in culture or using the UAS-Gal4 system (Brand and Perrimon, 1993) *in vivo* in the somatic follicle cells that surround the germline in the fly ovaries. To analyse the dynamic subcellular localisation of GFP–PigsFL, we imaged *Drosophila* tissue culture cells live and found that, when expressed at low levels, GFP–PigsFL localised to small comet-like structures (Fig. 1B,C). Coexpression of GFP–PigsFL with mCherry–Tubulin confirmed that GFP–PigsFL was localised to the ends of MTs (Fig. 1B), and indeed GFP–PigsFL partially colocalised with the plus-end-tracking protein (+TIP) EB1–mRFP when coexpressed (Fig. 1C). Time-lapse analysis of *Drosophila* tissue culture cells revealed that the GFP–PigsFL comets were motile (Fig. 1D; Movie 1), and moved with a median speed of 16.19 μm/min (Fig. 1E,F).

We next investigated whether Pigs showed a similar MT plus-end localisation *in vivo* in fly tissues. We expressed GFP–PigsFL in the somatic cells of the fly ovary because ovaries are amenable to short-term culture and live-imaging. Female ovaries are composed of egg-producing ovarioles, in which the germline is surrounded by an epithelial layer of somatic follicle cells (Fig. 1G). During the later stages of oogenesis (stage 9 or 10), the follicle cells that overlay the nurse cells of the germline flatten and become squamous (bright green in Fig. 1G), and are thus accessible for time-lapse imaging. In these cells, GFP–PigsFL showed a localisation to comets (arrows in Fig. 1H; Movie 2), but it also strongly localised to structures near the cortex of all cells. The time-lapse analysis revealed that the comet-like structures were motile (Fig. 1I,J), and moved at a median speed of 11.23 μm/min (Fig. 1M). This median speed is slightly lower than the one observed in tissue culture cells, but is comparable to comets labelled using the MT +TIP mCherry–CLIP170 (11.51 µm/min; see below). To determine whether GFP–PigsFL comets labelled the ends of MT *in vivo* we coexpressed mCherry–CLIP170 in the squamous follicle cells (Fig. 1K,L). When overexpressed as a tagged protein in fly tissues, human CLIP170, the orthologue of *Drosophila* CLIP190, concentrates at MT plus-ends but also labels the MT lattice (Stramer et al., 2010). Kymographs showed GFP–PigsFL tracked the MT plus-ends together with mCherry–CLIP170 (Fig. 1L). In order to analyse Pigs protein localisation at endogenous expression levels in a tissue where the protein is endogenously expressed, we generated flies containing a GFP-tag inserted at the endogenous genomic Pigs locus (PigsGFP^genomic^; see Materials and Methods). This PigsGFP^genomic^ was expressed at low levels in many tissues (Fig. S1), consistent with the mRNA expression analysis (Celniker et al., 2009), but showed relatively strong expression in the imaginal ring cells of the third-instar larval salivary glands (Fig. S1A). Here, Pigs localised to the basal sides of the epithelial cells that form the tube of the gland (Fig. S1B). MTs in many epithelial cells are nucleated apically, thus extending dynamic plus-ends towards the basal side of the cell (Bartolini and Gundersen, 2006), and this has also been shown to be true for salivary glands at the end of embryogenesis (Myat and Andrew, 2002; Booth et al., 2014). Thus, the basal localisation of endogenous Pigs–GFP at the basal side of these epithelial cells supports the hypothesis that Pigs at endogenous protein levels is an MT +TIP *in vivo*.

### Pigs tracks MT plus-ends through one of its three Sx(I/L)P motifs

**Fig. 2.**
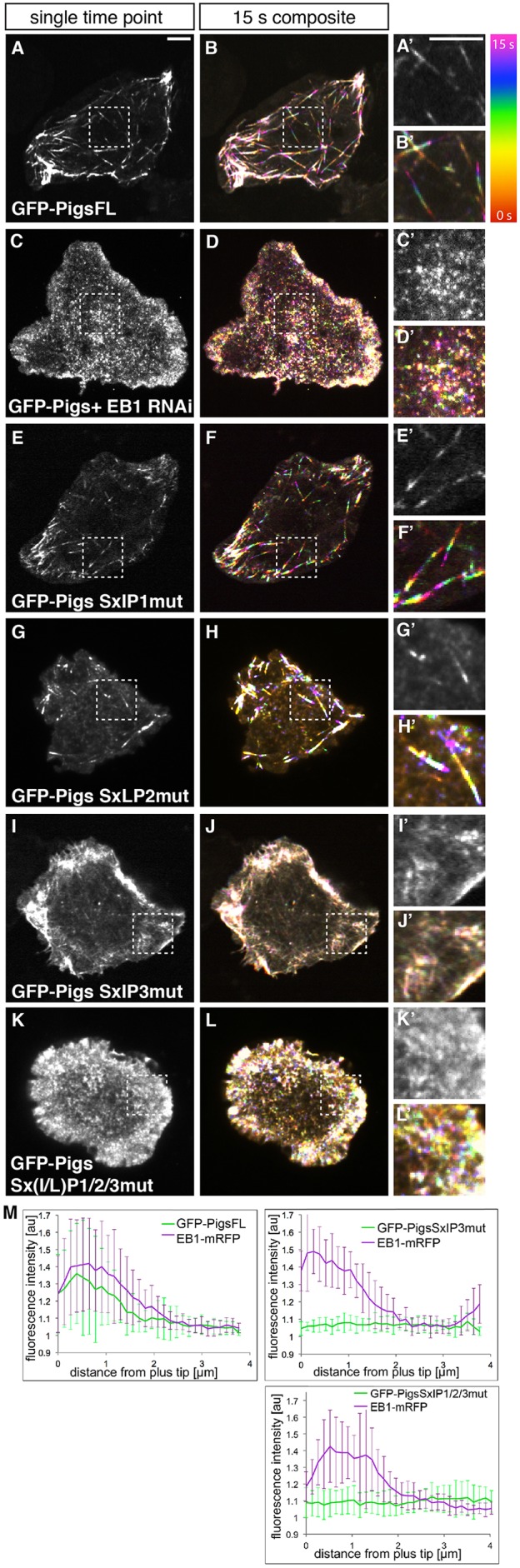
**One Sx(I/L)P motif mediates most MT plus-end tracking in tissue culture cells.** S2 cells expressing different GFP-tagged Pigs constructs were imaged live. Left panels are single frames from a time-lapse, right panels are maximum intensity projections of pseudocoloured frames from time-lapse movies to make a 15s composite image, with six images taken at 3 s intervals. Boxes indicate comets that are shown magnified to the right. (A,B) Wild-type GFP–PigsFL tracks MT plus-ends. See Movie 1. (C,D) Plus-end tracking is largely abolished in S2 cells treated with RNAi against EB1. See Movie 3. (E,F) GFP–PigsFL with Sx(I/L)P1 mutated (SxIP1mut) still tracks MT plus-ends. (G,H) GFP–PigsFL with Sx(I/L)P2 mutated (SxLP2mut) still tracks MT plus-ends. (I,J) GFP–PigsFL with Sx(I/L)P3 mutated (SxIP3mut) has lost the ability to track MT plus-ends. See Movie 4. (K,L) Mutation of all 3 SxIP motifs in GFP–PigsFL [Sx(I/l)P1/2/3mut] abolishes plus-tip tracking. See Movie 5. Scale bar is 5 μm in A and A', and applies to all images. (M) Quantitative line scans of MT plus ends performed on individual time frames of movies obtained from live cells coexpressing EB1–RFP and GFP–PigsFL (16 comets from 7 cells), GFP–PigsSxIP3mut (9 comets from 3 cells) or GFP–PigsSxIP1/2/3mut (14 comets from 4 cells). Results are mean±S.D.

As Pigs displayed stereotypical plus-end tracking behaviour both *in vivo* and in tissue culture cells, we next sought to determine how Pigs localises to the MT plus-end. There are three core MT +TIP families: end-binding proteins (EBs), the CAP-Gly-containing proteins [CLIP170/CLIP190 and p150^-glued^ (also known as DCTN1)], and TOG-domain-containing proteins, which include human Ch-TOG (also known as CKAP5), *Xenopus* XMAP215, and *Drosophila* Mini-spindles. Most +TIPs bind one of these core proteins as a means of being targeted to the plus-end (Akhmanova and Steinmetz, 2008). When looking at the amino acid sequence of the C-terminus of Pigs, we observed several potential Sx(I/L)P motifs, motifs that can mediate interaction with end-binding proteins. To test whether the MT plus-end localisation of GFP–PigsFL was EB1 dependent, we knocked down EB1 using RNA interference (RNAi) in S2 cells and observed GFP–PigsFL localisation in cells. We found that, although MTs were present, the GFP–PigsFL comets were lost (Fig. 2A,B versus C,D; Movie 3) indicating that Pigs relied on an association with EB1 for its plus-end tracking, and that this association is likely through its Sx(I/L)P motifs.

Upon closer analysis of the C-terminus of Pigs we identified three potential Sx(I/L)P motifs [S678–P681 (SxIP1), S712–P715 (SxLP2) and S964–P967 (SxIP3)] (Fig. 1A). We next individually mutated the isoleucine or leucine, and proline residues of each Sx(I/L)P motif (I680N and P681N in SxIP1, L714N and P715N in SxLP2, and I966N and P967N in SxIP3) in GFP-tagged full-length Pigs, and observed the dynamics and subcellular localisation. We found that mutation of SxIP1 or SxLP2 had little effect on the MT plus-end localisation of full-length Pigs (Fig. 2E–H′), and the plus-end-tracking behaviour could still be observed throughout the cell in time-lapse movies (Fig. 2F,H). Mutation of SxIP3, however, eliminated MT plus-end-tracking in tissue culture cells (Fig. 2I–J′) as well as *in vivo* (Movie 4). Consistent with these results, mutation of all three sites also completely abolished it (Fig. 2K–L′; Movie 5). The loss of plus-end-tracking ability was also obvious from intensity scans along MT plus-ends in live cells coexpressing EB1–mRFP and GFP–PigsFL, GFP–PigsSxIP3mut or GFP–PigsSxIP1/2/3mut, with the former being concentrated near the tip with EB1, and the latter two being evenly distributed along MT tip and shaft (Fig. 2M). It, therefore, seems that the EB1-dependent plus-end tracking behaviour of Pigs depends mainly on Sx(I/L)P3.

### Pigs can bind MTs and actin filaments in cultured cells and *in vivo*

**Fig. 3.**
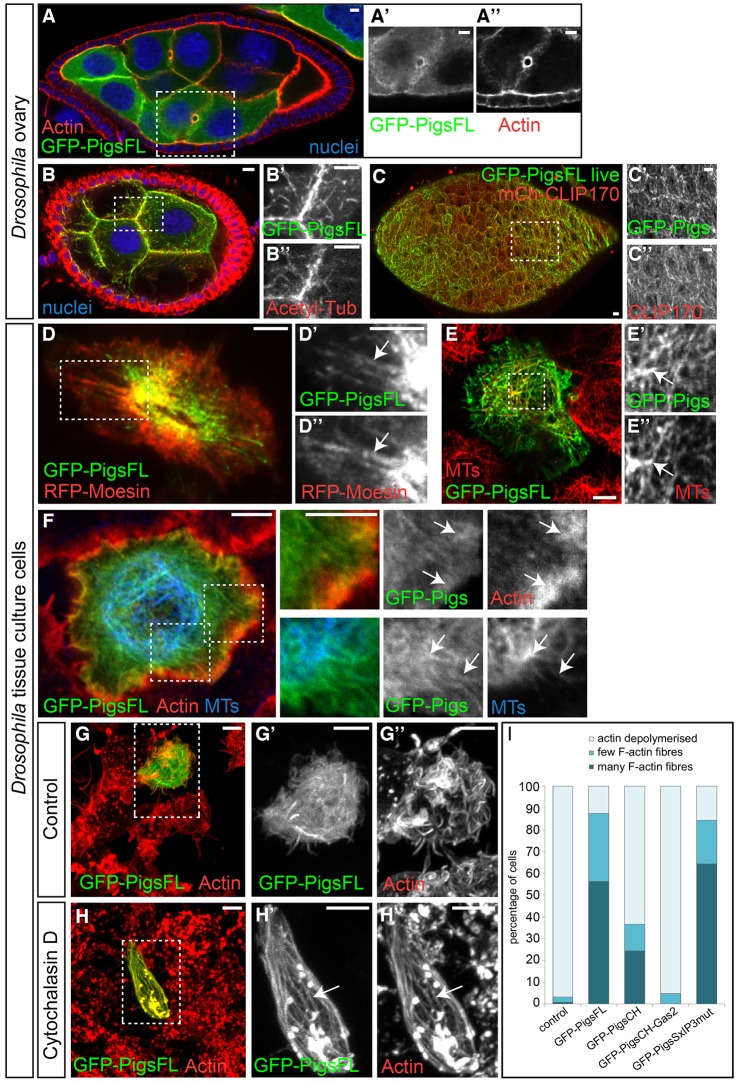
**Full-length Pigs colocalises with MTs and actin networks *in vivo* and *in vitro.*** (A) GFP–PigsFL (green, A′) expressed in the germline of the *Drosophila* ovary at stage 8 colocalises with actin-rich ring canals (red, A″). Nuclei, blue. (B) GFP–PigsFL (green, B′) also colocalises with MTs (red, B″) in the ovarian germline at stage 6. (C) GFP–PigsFL (green, C′) colocalises with MTs (red, C″) in the ovarian follicle cells at stage 8. (D) GFP–PigsFL (green, D′) partially colocalises with actin marked by RFP–Moesin–ABD (RFP-Moesin, red, D′) in S2 cells. (E) GFP–PigsFL (green, E′) partially colocalises with MTs labelled with α-tubulin (red, E″) in S2R+ cells. (F) GFP–PigsFL (green) at elevated expression levels partially colocalises with actin (phalloidin, red) and MTs (anti-acetylated-tubulin, blue) in S2R+ cells. Arrows in D–F highlight regions of colocalisation. (G) S2R+ cells treated with DMSO and stained with phalloidin to label actin (red, G″). One cell expresses GFP–PigsFL (G,G′). (H) S2R+ cells treated with the actin-depolymerising drug cytochalasin D for 15 min stained with phalloidin to label actin (red). GFP–PigsFL (green, H′) binds to and protects much of the actin (red, H″) from depolymerisation. The arrow highlights colocalisation on fibrous structures. Panels indicated by primes show magnifications of the boxed region. Scale bars: 5 μm. (I) Quantification of the protective effect of GFP–Pigs expression on F-actin in preventing depolymerisation by cytochalasin D; indicated constructs were analysed. The chart shows the effects for cells of medium expression levels (analysis of does-dependence on expression level is shown in Fig. S2). The following numbers of cells were counted from three experiments: control (*n*=121); GFP-PigsFL (*n*=144); GFP-PigsCH (*n*=126); GFP-PigsCH-Gas2 (*n*=53); GFP-PigsSxIP3mut (*n*=153).

The above results demonstrate that Pigs is an EB1-dependent plus-end-tracking protein. We had previously observed that full-length Pigs colocalised with both MTs along their length and with some actin structures when it was expressed in the ovary *in vivo* (Pines et al., 2010). Here, we confirmed that at elevated expression levels in the germline of the ovary, in addition to plus-end tracking, GFP–PigsFL also colocalised with both the actin-rich ring canals that connect nurse cells (Fig. 3A) and MT shafts (Fig. 3B). When expressed in follicle cells of the ovaries together with mCherry–CLIP170 and imaged live, GFP–PigsFL predominantly showed a high-level of colocalisation with MTs (Fig. 3C). This difference in preferential localisation to actin and MTs versus MTs alone suggests that Pigs might serve different functions depending on the tissue and stage of development. Similarly, GFP–PigsFL expressed in *Drosophila* tissue culture cells showed colocalisation with the actin labels RFP-tagged Moesin actin-binding domain (Moesin-ABD) (Fig. 3D) and phalloidin (Fig. 3F) in some cells, and with MTs in others (Fig. 3E,F).

One potential effect of cytolinkers binding to cytoskeletal structures is their stabilisation. To determine whether GFP–PigsFL expression affected actin stability, we treated cells with the depolymerising drug cytochalasin D. In control cells, GFP–PigsFL only partially colocalised with the actin cytoskeleton (Fig. 3G). However, upon cytochalasin D treatment, the amount of F-actin remaining in the GFP–PigsFL-expressing cells was much greater than in the surrounding cells, and GFP–PigsFL strongly colocalised with these actin fibres (Fig. 3H). This indicates that GFP–PigsFL can bind to and protect actin from depolymerisation by cytochalasin D.

**Fig. 4.**
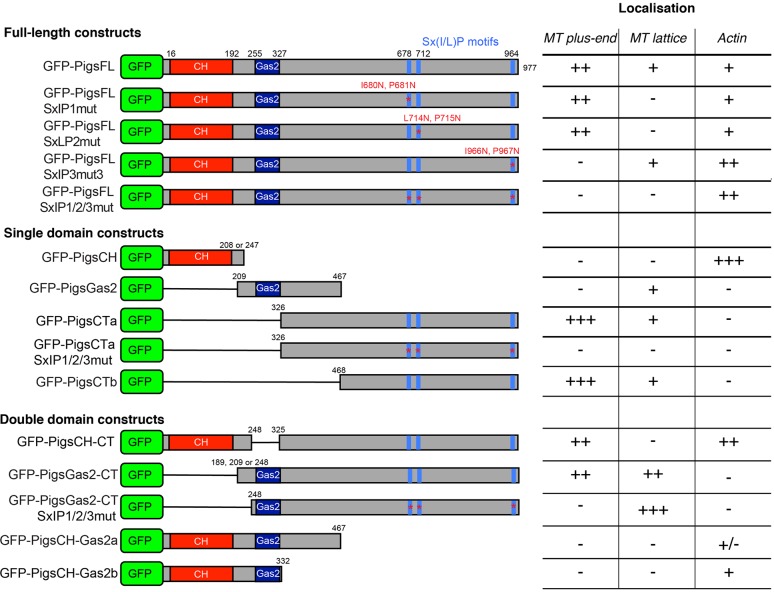
**Pigs expression constructs.** Schematic of the different Pigs expression constructs used in this study. The positions of the N-terminal GFP tag, the CH and Gas2 domains and Sx(I/L)P motifs are indicated. Numbers above the schematics indicate the amino acid position of the boundary of a particular domain or motif. The table on the right summarises the ability of the different constructs to interact with actin, MT lattice and MT plus-ends, as identified both in tissue culture cells *in vitro* as well as in *Drosophila* tissues *in vivo*. −, no colocalisation; +/−, occasional colocalisation; +, ++ and +++; increasing amount of colocalisation.

The results above show that full-length Pigs has a complex localisation pattern, which is likely the result of a combination of its cytoskeletal-interacting domains. We therefore wanted to dissect in detail which domains of Pigs were responsible for these different aspects. We split Pigs into three separate regions: the CH domain that potentially binds actin, the Gas2 domain that potentially interacts with MTs, and the C-terminal half that contains the plus-end-tracking Sx(I/L)P motifs and many positively charged amino acids that could also interact with MTs. We first analysed GFP fusion constructs consisting of the single regions to identify whether, in isolation, these regions can interact with the cytoskeleton. Then we tested combinations of these regions to determine whether there was any interaction between them (constructs analysed are outlined in Fig. 4).

### The CH domain mediates the localisation of Pigs to actin structures

**Fig. 5.**
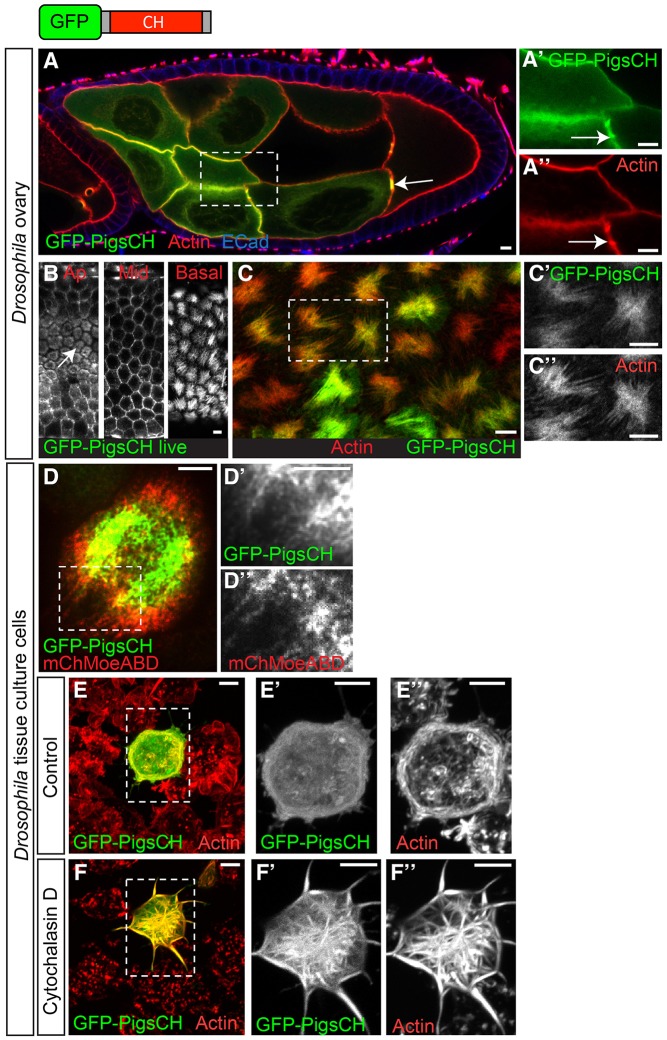
**The single CH domain of Pigs mediates binding to actin.** (A) When GFP–PigsCH (green, A′) is expressed in the germline of the *Drosophila* ovary at stage 8 it strongly colocalises with actin stained by phalloidin (red, A″), both at the cortex and ring canals (arrows). E-Cadherin (ECad), blue. (B) Live imaging of GFP–PigsCH expressed in the ovary follicle cells. GFP–PigsCH localises to actin-rich structures at all apico-basal levels: microvilli at the apical surface (arrow), the cortex at mid levels, and the stress fibre-like structures at basal levels. (C) Phalloidin staining of ovary follicle cells expressing GFP–PigsCH (green, C′) reveals colocalisation of GFP–PigsCH with actin (red, C″). (D) S2 cell coexpressing GFP–PigsCH and mCherry–Moesin-ABD (mChMoeABD) shows GFP–PigsCH partially colocalises with actin. (E) S2R+ cells treated with DMSO and stained with phalloidin to label actin (red, E″). GFP–PigsCH (green, E′) partially colocalises with actin. (F) S2R+ cells treated with the actin-depolymerising drug cytochalasin D for 15 min and stained with phalloidin to label actin (red, F″). GFP–PigsCH binds to and protects the actin (red, F″) from depolymerisation. Panels indicated by primes show magnifications of the boxed region. Scale bars: 5 μm.

Because the N-terminus of Pigs contains a CH domain that is hypothesised to bind actin (Fig. 1A), we tested whether it mediates actin binding in Pigs. Expression of only the CH domain of Pigs fused to GFP (GFP–PigsCH) in the germline cells of the fly ovary showed a strong localisation to the actin-rich ring canals and the cell cortex (Fig. 5A). Live imaging of GFP–PigsCH in the follicle cells showed that GFP–PigsCH localised to actin-rich structures throughout the cell (Fig. 5B), namely the microvilli at the apical surface (arrow in Fig. 5B), the actin cortex at mid apico-basal levels and the basal stress-fibre like structures. Staining of the ovaries with phalloidin to label actin confirmed a strong colocalisation of GFP–PigsCH with the basal actin (Fig. 5C). Furthermore, when we expressed GFP–PigsCH in S2 cells, this construct appeared to incorporate into the actin cytoskeleton in a filamentous manner (Fig. 5D). In fact, in many cells, GFP–PigsCH colocalised with the Moesin-ABD (Fig. 5D). Consistent with its localisation, we found that the CH domain of Pigs conferred the actin-stabilising ability of Pigs. Similar to the behaviour of the full-length GFP–Pigs, GFP–PigsCH only partially colocalised with actin in control S2R+ cells (Fig. 5E), but when treated with cytochalasin D, the actin was protected from depolymerisation in cells expressing GFP–PigsCH, and localisation of GFP–PigsCH to the actin structures was enhanced (Fig. 5F).

### The Gas2 domain can bind to MTs

**Fig. 6.**
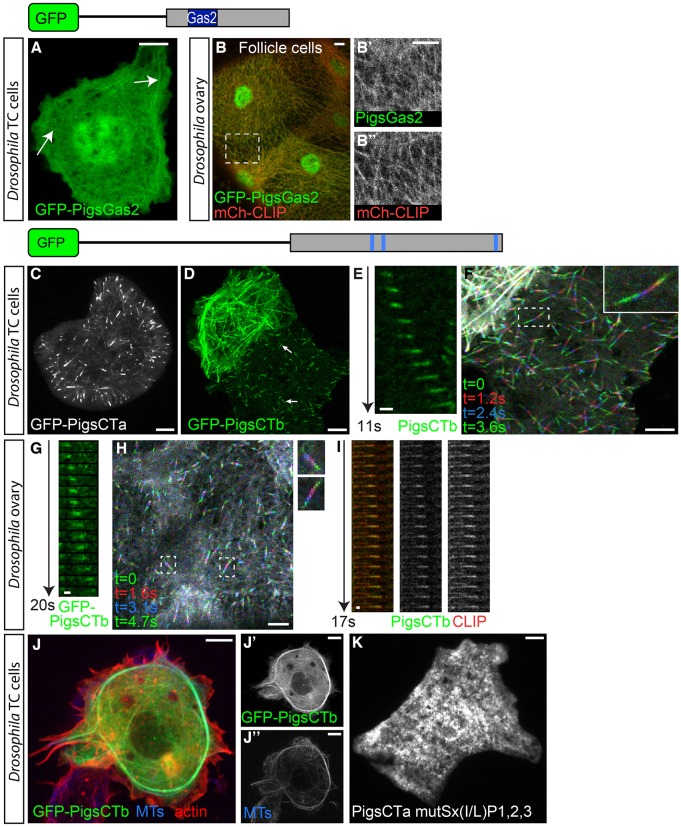
**Interaction of Pigs with MTs.** Pigs interacts with MTs in two different ways: the Gas2 domain localises to the MT lattice and the C-terminal half of Pigs is responsible for MT plus-end tracking. (A) S2R+ cell expressing GFP–PigsGas2 imaged live. GFP–PigsGas2 shows weak lattice localisation reminiscent of MTs (arrows). (B) Live imaging of GFP–PigsGas2 (green, B′) and mCherry–CLIP170 (mCh-CLIP; red, B″) expressed in the ovary squamous follicle cells at stage 10. GFP–PigsGas2 colocalises with the MTs labelled with mCh-CLIP. Box indicates magnified area shown in B′ and B″. (C) Live imaging of an S2 cell expressing GFP–PigsCTa reveals a comet-like localisation to MT plus-ends. (D) Live imaging of two S2R+ cells expressing GFP–PigsCTb. At low levels GFP–PigsCTb shows a comet-like localisation (arrows in lower cell), but at higher levels shows a lattice localisation (upper cell). See Movie 6. (E) Kymograph of GFP–Pigs tracking an MT plus-end in a S2R+ cell over 11 s. (F) Frames from a time-lapse movie of a S2R+ cell expressing GFP–PigsCTb shown as a maximum intensity projection of four consecutive frames pseudocoloured in green (*t*=0 s), red (*t*=1.2 s), blue (*t*=2.4 s), and then green (*t*=3.6 s) again. The inset shows a magnified single comet. (G) Kymograph of GFP–PigsCTb tracking an MT plus-end in the ovary squamous follicle cells over 20 s. (H) Maximum intensity projection of pseudocoloured frames from a time-lapse movie of ovarian squamous follicle cells at stage 10 expressing GFP–PigsCTb. Boxes indicate single comets shown magnified to the right. (I) Kymograph of GFP–Pigs (green) and mCherry–CLIP170 (red) tracking an MT plus-end in the ovary over 17 s. (J) An S2R+ cell overexpressing GFP–PigsCTb (green, J′) induces bundling of MTs labelled by anti-α-tubulin antibody (blue, J″) cells. Actin labelled by phalloidin is in red. J′–J″ show the separate channels for GFP and MTs. (K) PigsCTa with all 3 Sx(I,L)P sites mutated loses all MT localisation. Scale bars: 5 μm (A–D,F,H,J,K); 1 μm (E,G,I).

In some cells and tissues, full-length Pigs localised to the MT lattice. To test whether the Gas2 domain of Pigs was responsible for the MT shaft binding, we expressed this domain fused to GFP (GFP–PigsGas2). Live imaging of S2R+ cells expressing GFP–PigsGas2 revealed a weak localisation to fibres that were likely to be MTs (arrows in Fig. 6A). When coexpressed with mCherry–CLIP170 in the squamous follicle cells at stage 10 and imaged live, we observed a strong colocalisation of GFP–PigsGas2 with the MTs labelled by mCherry–CLIP170 (Fig. 6B). Interestingly, the interaction of GFP–PigsGas2 with MTs was lost upon fixation (data not shown) in both cells and tissues, suggesting that the interaction with MTs was weak.

### The C-terminal half of Pigs contains Sx(I/L)P motifs and tracks MT plus-ends

We have shown above that one of the Sx(I/L)P motifs in the context of the full-length protein mediated MT plus-end tracking in cells in culture. However, it is not uncommon for plus-end-tracking proteins to interact with the MT plus-end through several different mechanisms (Honnappa et al., 2009; Akhmanova and Steinmetz, 2010; Applewhite et al., 2010). To investigate whether the Sx(I/L)P motifs were sufficient to mediate the plus-end tracking, we expressed a construct containing all three motifs of Pigs fused to GFP (GFP–PigsCT). Live imaging of *Drosophila* tissue culture cells expressing GFP–PigsCT indeed showed that, at low expression levels, GFP–PigsCT was almost exclusively localised to comets (Fig. 6C,D; Movie 6) and time-lapse imaging confirmed that the comets were motile (Fig. 6E,F) with an median speed of 16.64 μm/min (Fig. 1F). This strongly suggests that GFP–PigsCT can track MT plus-ends. We observed a similar behaviour of GFP–PigsCT *in vivo* when expressed in the squamous follicle cells (Fig. 6G,H), and time-lapse analysis of GFP–PigsCT coexpressed with mCherry–CLIP170 confirmed that GFP–PigsCT tracked the MT plus-ends (Fig. 6I). The speed of GFP–PigsCT comets *in vivo* averaged 11.72 μm/min (Fig. 1M), slightly lower than the speed of GFP–PigsCT comets in *Drosophila* tissue culture cells. When expressed at elevated levels, GFP–PigsCT was able to bundle MTs, an ability only rarely observed for expression of the full-length protein (Fig. 6J).

As described above, depending on the expression level, the C-terminal half of Pigs, containing the Sx(I/L)P motifs (GFP–PigsCT), not only interacted with MTs at their plus-ends, but also showed binding along the MT shaft. We therefore investigated whether both modes of MT localisation were dependent on the Sx(I/L)P motifs or whether other parts of the C-terminal half of Pigs played a role in MT association. We found that the CT-terminal half of Pigs, with all three Sx(I/L)P motifs mutated, did not localise to MT lattice, rather it appeared cytoplasmic (Fig. 6K), suggesting that in the absence of both the Gas2 domain and functional Sx(I/L)P motifs, Pigs is unable to associate with MTs.

### In the absence of the CH domain, the Gas2 domain can mediate MT lattice interactions, even when Sx(I/L)P motifs are mutated

**Fig. 7.**
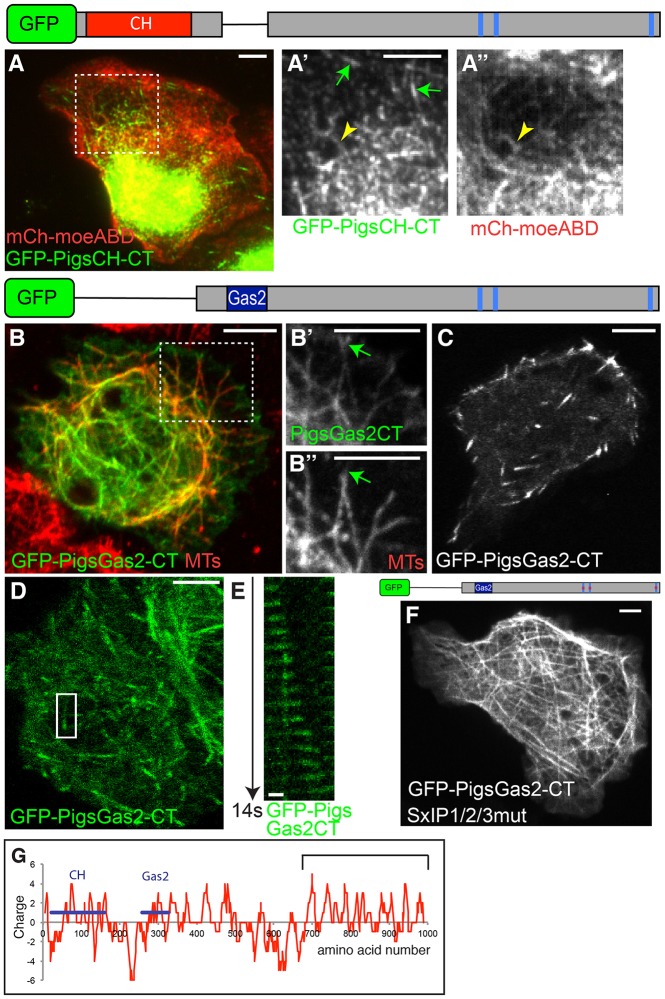
**The C-terminus of Pigs imposes dominant MT plus-end tracking in S2R+ cells and *in vivo*.** (A–A″) Live imaging of an S2 cell coexpressing GFP–PigsCH-CT (green) and the actin label mCherry–Moesin-ABD (mCh-moeABD; red). GFP–PigsCH-CT shows both a comet-like localisation (green arrows) and a partial colocalisation with actin (arrowhead). (B–B″) S2R+ cell expressing GFP–PigsGas2-CT (green, B′) stained for MTs using anti-α-tubulin antibody (red, B″). GFP–PigsGas2-CT localises both to MT plus-ends (arrows) and the MT lattice. The boxed area is shown magnified on the right. (C) Live imaging of a S2 cell expressing GFP-PigsGas2-CT showing a comet-like localisation. (D) Live imaging of a S2R+ cell expressing GFP-PigsGas2-CT. Box shows position of tracked comet. See Movie 7. (E) Kymograph of GFP–PigsGas2CT tracking an MT plus-end in a S2R+ cell over 14 s. (F) GFP–PigsGas2-CT with all Sx(I/L)P sites mutated localises to the MT lattice. (G) Charge analysis of Pigs showing a concentration of positively charged amino acids in the C-terminal third of the protein (bracket). Scale bars: 5 μm (A–D,F); 1 μm (E).

To investigate whether the CH or Gas2 domains affected the ability of the C-terminal half of Pigs to track MT plus-ends, we expressed constructs consisting of the C-terminal half with either the CH (GFP–PigsCH-CT) or Gas2 domain (GFP–PigsGas2CT) in cultured cells. We found that GFP–PigsCH-CT localised partly to comets, but also showed some colocalisation with actin (Fig. 7A), consistent with the domains present. In fixed cells stained for MTs, GFP–PigsGas2CT colocalised with the MT lattice, but could also be seen concentrated at the MT plus-ends (Fig. 7B). Live imaging revealed that, in some cells, GFP–PigsGas2CT concentrated at just the MT plus-ends (Fig. 7C,D; Movie 7), and the kymograph illustrates the behaviour of a single comet from a time-lapse movie (Fig. 7E). The median speed of GFP–PigsGas2CT comets in *Drosophila* tissue culture cells was 17.08 μm/min (Fig. 1F).

Similar to the PigsCT construct, PigsGas2-CT not only interacted with MTs at their plus-ends, but it also showed binding along the MT shaft. However, when we expressed GFP-tagged PigsGas2-CT with all Sx(I/L)P motifs mutated, we observed a clear shift to lattice association, suggesting that the Gas2 domain is mediating this interaction (Fig. 7F). Interestingly, this lattice localisation is stronger compared to that mediated by the Gas2 domain alone, indicating that other factors in the C-terminal region might aid the shaft association. The C-terminal half of Pigs contains many positively charged amino acids that could interact with the negatively charged MTs and might explain this association (Fig. 7G).

### The CH domain and Gas2 domain have mutually inhibitory effects on cytoskeleton binding

**Fig. 8.**
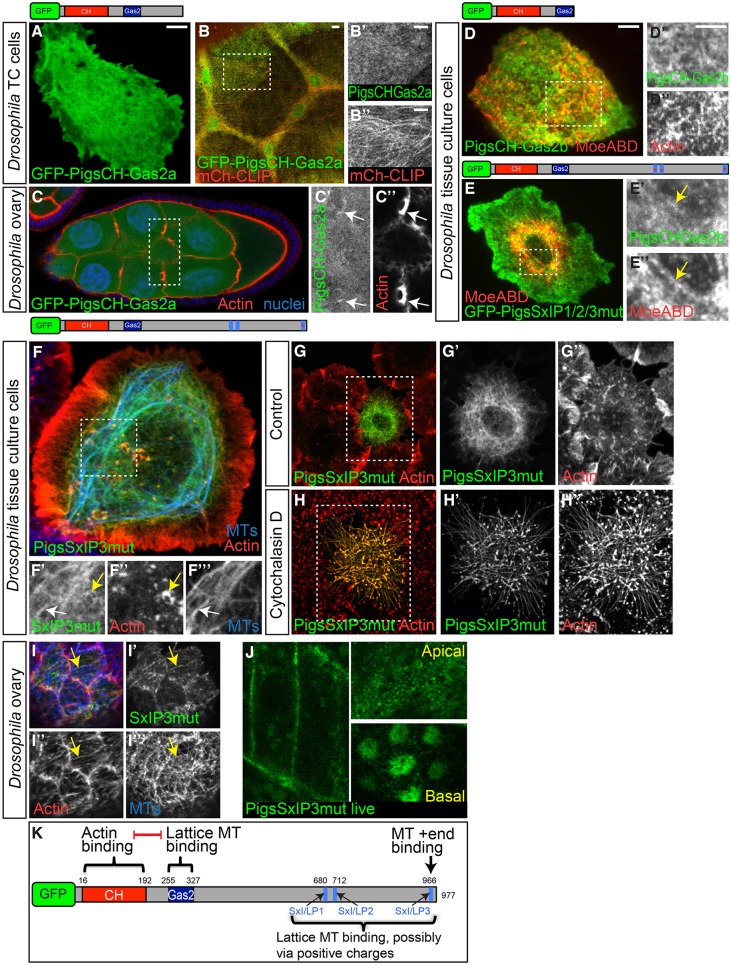
**The CH and Gas2 domains of Pigs mutually negatively affect cytoskeleton interaction.** (A) S2R+ cell expressing GFP–PigsCH-Gas2a imaged live. GFP–PigsCH-Gas2a is diffusely localised in the cytoplasm. (B) Live imaging of GFP–PigsCH-Gas2a (green, B′) and mCherry–CLIP170 (mCh-CLIP; red, B″) expressed in the ovary squamous follicle cells at stage 10. GFP–PigsCH-Gas2a does not colocalise with MTs. (C) When GFP-PigsCH-Gas2a (green, C′) is expressed in the germline of the *Drosophila* ovary at stage 9 it only weakly colocalises with ring canals (arrows) that are rich in actin labelled using phalloidin (red, C″), most is cytoplasmic. (D) Live imaging of an S2 cell coexpressing GFP–PigsCH-Gas2b (D′) and mCherry–Moesin-ABD (MoeABD; D″), illustrating that in some cells GFP–PigsCH-Gas2b can partially colocalise with actin. (E) Live imaging of an S2 cell coexpressing GFP–PigsSxIP1/2/3mut (E′) and mCherry–Moesin-ABD (MoeABD, E″), illustrating that GFP–PigsSxIP1/2/3mut partially colocalises with actin. (F) GFP–PigsSxIP3mut (green, F′) colocalises with both actin (red, F″) and MTs (blue, F‴) in S2R+ cells. Arrows in E and F highlight regions of colocalisation. (G) S2R+ cells treated with DMSO and stained with phalloidin to label actin (red, G″). One cell expresses GFP–PigsSxIP3mut (G,G′). (H) S2R+ cells treated with the actin-depolymerising drug cytochalasin D for 15 min and stained with phalloidin to label actin (red). GFP–PigsSxIP3mut (green, H′) binds to and protects much of the actin (red, H″) from depolymerisation. Panels indicated by primes show magnifications of the boxed region. (I–I‴) GFP–PigsSxIP3mut (green, I′) *in vivo* in fixed ovarian follicle cells localises to both actin (red, I″) and MTs (blue, I‴) structures and often colocalises with both (arrow). (J) GFP–PigsSxIP3mut imaged live in ovarian follicle cells strongly labels actin-based structures, such as the cortical actin of the follicle cells, apical microvilli and basal stress-fibre-like arrays. (K) Summary diagram of the functions of the different domains of Pigs and their interactions with each other. Scale bars: 5 μm.

The PigsCT-Gas2 construct with the Sx(I/L)P sites mutated localised to the MT lattice. However, the full-length protein without Sx(I/L)P sites (although containing Gas2 and CH domains) only localised to actin structures, suggesting that the CH domain has an inhibitory effect on the ability of the Gas2 domain to bind MT shafts. To investigate this potential functional interplay between these two modes of cytoskeletal association further, we analysed the localisation of Pigs constructs containing both of these domains (termed GFP–PigsCH-Gas2a/b). When expressed and imaged live in fly tissue culture cells or in the squamous follicular epithelium, GFP–PigsCH-Gas2a had a largely cytoplasmic distribution (Fig. 8A,B). In the fly germline, GFP–PigsCH-Gas2a showed some cytoskeletal binding as it partially colocalised with the phalloidin-labelled ring canals of nurse cells (Fig. 8C). However, a large amount of GFP–PigsCH-Gas2a was diffusely distributed within the cytoplasm, and no labelling of MTs could be observed (Fig. 8B). When we expressed GFP–PigsCH-Gas2b in fly tissue culture cells, we observed that this construct could weakly incorporate into the actin cytoskeleton and did not appear to associate with the MT cytoskeleton to any extent (Fig. 8D).

A second way of comparing the CH with the Gas2 domain in the context of the whole protein is to eliminate plus-end tracking ability, which is the other functional cytoskeleton interaction domain. Plus-end tracking was equally reduced by mutating either the third or all three SxIP motifs in the full-length protein (Fig. 2), and thus we analysed the cytoskeletal localisation of both of these constructs more closely. We found that GFP–PigsSxIP1/2/3mut partially colocalised with actin in *Drosophila* tissue culture cells (Fig. 8E), similar to GFP–PigsCH-Gas2b. GFP–PigsSxIP3mut partially colocalised with actin and also with MT shafts (Fig. 8F). Upon treatment with cytochalasin D, GFP–PigsSxIP3mut was able to protect actin from depolymerisation to a similar extent to GFP–PigsFL (Figs 3I and 8H) and the actin fibres were more pronounced compared to control DMSO-treated cells (Fig. 8G). Finally, we investigated the localisation of GFP–PigsSxIP3mut *in vivo*, where live imaging revealed that, depending on the tissue context, it colocalised with both actin and MTs – at the basal surface of follicle cells of stage 8 egg chambers GFP–PigsSxIP3mut highlighted both actin and MTs (Fig. 8I), and it also strongly labelled the actin cortex, apical microvilli and stress-fibres in follicle cells at stage 10 (Fig. 8J).

In summary, the weak cytoskeletal localisation of GFP–PigsCH-Gas2a/b to actin-based structures only, in comparison to the strong actin localisation displayed by the GFP–PigsCH construct (Fig. 5A–C), and to the MT-binding ability displayed by the Gas2 domain alone (Fig. 6B), suggests that the presence of the Gas2 domain has an inhibitory effect on the ability of the CH domain of full-length Pigs to bind to actin. This was also evident from the inability of GFP–PigsCH-Gas2a to protect actin from depolymerisation by cytochalasin D, whereas GFP–PigsCH acted protectively (Fig. 3I; Fig. S2C,D). Vice versa, the presence of the CH domain interfered with the ability of the Gas2 domain to interact with MTs. The enhanced ability of GFP–PigsSxIP3mut compared to GFP–PigsFL to localise to actin and MT-based structures *in vivo* suggests that plus-end tracking also interferes with the ability of the CH- and the Gas2-domains to interact with actin and MTs, respectively.

It will be important to determine in the future, how these intramolecular regulatory effects are modulated by further interaction partners as well as subcellular localisation within the cell.

## DISCUSSION

Cytolinker proteins play important roles in many processes during development and tissue homeostasis. Although most cytolinkers contain clearly identifiable domains predicted to mediate the interaction with actin or MTs, *in vivo* analysis has often demonstrated that cytoskeletal association and crosslinking is a multi-layered and regulated process. In the case of the single *Drosophila* Gas2-like cytolinker Pigs, we have uncovered a complex regulation of binding to MTs as well as actin. Although we found Pigs to predominantly be a bona fide MT plus-end tracker, mediated through EB1-binding by one of its three Sx(I/L)P motifs, at elevated protein levels MT shaft binding was also observed. Furthermore, the analysis of individual or paired domains revealed a mutual inhibitory relationship between the CH and Gas2 domains, as well as a decreased ability of CH and Gas2 domains to bind actin and MTs when Sx(I/L)P motifs and plus-end tracking is present.

Is Pigs, in addition to being an MT plus-end tracker, also a bona fide cytolinker? Full-length Pigs localises most extensively to MT plus-ends, as well as the shaft depending on expression levels, but, especially *in vivo* in fly tissues, it colocalises with both actin and MTs near cell cortices. Moreover, Pigs can protect actin from drug-induced deploymerisation, and, upon depolymerisation, GFP–Pigs and protected actin colocalised with MTs (data not shown). These features are all shared with the spectraplakin cytolinker Shot. We have previously described a role for an intramolecular association leading to regulated inhibition of the cytoskeletal crosslinking ability of Shot – Shot tracks MT plus-ends in a folded back inhibited conformation that is released and allows crosslinking activity between MT shafts and actin near cortical areas (Applewhite et al., 2013). The dynamic behaviour of Pigs, as well as the mutual inhibitory relationship between the CH and Gas2 domains, suggests that a related mechanism could be at work in the case of Pigs, possibly also allowing the crosslinking activity of Pigs to be targeted to specific subcellular localisations.

The functional analysis of Pigs is complicated by the fact that some of its function might be redundant with other cytolinkers, in particular Shot. Shot has many well-identified roles from embryogenesis into adulthood, as well as during oocyte specification (Gregory and Brown, 1998; Lee et al., 2000; Röper et al., 2002; Röper and Brown, 2003). *pigs^1^* mutants are viable but infertile (Pines et al., 2010). Both Shot and Pigs show a ubiquitous expression pattern, although Pigs appears to be expressed only at low levels in many tissues (Fig. S1). With Shot appearing to be the dominant developmental cytolinker of the CH-Gas2 class in flies, Pigs might add extra functionality under certain conditions, possibly regulated by its responsiveness to Notch (Pines et al., 2010), with loss of Pigs phenotypes masked by a partial redundancy with Shot in many instances.

It is tempting to speculate that the different arrangement of the CH and Gas2 domains and MT plus-end tracking Sx(I/L)P motifs in Shot compared to Pigs affects the functionality of these cytolinkers. The actin and MT interaction domains in all Shot isoforms are separated by a physical distance of at least 200 nm (potentially up to 400 nm in the largest Shot isoforms), whereas in Pigs these domains lie much closer together. Thus, the crosslinked cytoskeletal network stabilised by either cytolinker might well differ substantially in its arrangement. Use of superresolution microscopy and careful 3D reconstruction of cytoskeletal arrangements will help address this in the future.

Pigs is the sole Gas2-like family member in flies, compared to the vertebrate family of three Gas2-like proteins and the founding member Gas2 itself. Although Pigs appears to share features of cytoskeletal interaction with its vertebrate counterparts, where this has been analysed thus far, the shift from a single to multiple Gas2L proteins in vertebrates appears to have allowed divergence of function of these paralogues. All Gas2L proteins can interact with both actin and MTs, but only the isolated C-termini of Gas2L1 and Gas2L2 clearly colocalise with EB1 at MT plus-ends in mammalian tissue culture cells, whereas the overexpressed full-length proteins label the whole MT. In contrast to ectopic Pigs expression, overexpression of Gas2L1 and Gas2L2 strongly affects MT dynamics, probably because end-binding proteins become immobilised along the shaft (Stroud et al., 2014). Gas2L1 and Gas2L2 share with Pigs the very C-terminal positioning of a functional Sx(I/L)P motif, whereas Gas2L3 has two Sx(I/L)P-related motifs placed in the centre of the molecule, and its isolated C-terminus does not track plus-ends (Stroud et al., 2014). In contrast to Pigs, Gas2L3 also cannot protect actin from drug-induced depolymerisation (Stroud et al., 2011). Thus, Gas2L3 appears the most divergent of the group, and one of its characterised functions, as a target of the DREAM complex involved in cytokinesis (Wolter et al., 2012; Pe'er et al., 2013), is also a function not observed in Pigs or Shot, suggesting the expansion of the Gas2L family in vertebrates might have freed some members to evolve to take on new roles.

Molecular studies of Gas2L family members in vertebrates have thus far been near exclusively been performed in tissue culture cells (Wolter et al., 2012; Sharaby et al., 2014; Stroud et al., 2014). Our parallel use of *Drosophila* tissue culture cells as well as expression and analysis of Pigs in a variety of *Drosophila* tissues *in vivo* has revealed that, in many cases, localisation of Pigs variants to MT- or actin-based structures *in vivo* was much more pronounced than *in vitro*. In particular actin is arranged into many more complex structures in tissues *in vivo* than is found in tissue culture cells, with many of these arrangements being highly tissue specific. In addition, the overlap of such actin structures with the MT cytoskeleton might constitute an important aspect of cytoskeletal function in tissues. The nature of the cytoskeletal networks present in tissues *in vivo* clearly affects the localisation of Pigs, and its differential localisation highlights the need, as well as the distinct advantage, of an *in vivo* analysis of cytolinker function in intact tissues.

## MATERIALS AND METHODS

### Cloning of the GFP–Pigs constructs

GFP-tagged constructs for PigsFL, PigsNT, PigsCT, PigsCH, PigsGas2 and PigsGas2CT were amplified by PCR from the cDNA clone RE60364 [*Drosophila* Genomics Resource Center (DGRC), Bloomington, IN]. GFP–PigsFL was the same as previously described (Pines et al., 2010). GFP–PigsCH, GFP–PigsG2 and GFP–PigsG2CT Pigs constructs were cloned into the pUASp-GFP vector (Röper and Brown, 2003). GFP–PigsSxIP3mut was cloned by mutagenesis of PigsFL in pUASpGFP and then transferred to pUASTattB. The following primers were used to clone the different GFP–Pigs constructs: GFP–Pigs-FL, NT_PIGSfwd, 5′-CCCCCCCTCGAGATGGCCATGTTAGAGGCGCG-3′ and NT_PIGSrev, 5′-GGTAGGGCCCCTAGTAAAGCTCTGTATGATGACG-3′; GFP–Pigs-SxIPmut1, PIGSmSKIP1fwd, 5′-CCAACACCCAATCTCAGCAAGAACAACCGCTCTCCATTGGCG-3′ and PIGSmSKIP1rev, 5′-CGCCAATGGAGAGCGGTTGTTCTTGCTGAGATTGGGTGTTGG-3′; GFP–Pigs-SxIPmut2, PIGSmSKIP2fwd, 5′-GATTTGAGCAGCCGGTCTGGTAACAACGCTCCAGCTTTTAGC-3′ and PIGSmSKIP2rev, 5′-GCTAAAAGCTGGAGCGTTGTTACCAGACCGGCTGCTCAAATC-3′; GFP–Pigs-SxIPmut3, PIGSmSKIP3Fwd, 5′-CGAGAGAGGGGCATGTCCAAGAACAACGCGCCAGTGCGTCATCAT-3′ and PIGSmSKIP3rev, 5′-ATGATGACGCACTGGCGCGTTGTTCTTGGACATGCCCCTCTCTCG-3′; GFP–PigsCH, GFP-PigsCHup, 5′-AATCCGCGGCCGCTATGGCCATGTTAGAGGCG-3′ and GFP-PigsCHLow2, 5′-AATCTAGACTAGGCGGCACTACTATTTCC-3′; GFP–PigsGas2, GFP-PigsG2up, 5′-AAGCGGCCGCCACAATGACGACAATA-3′ and GFP-PigsGas2Low, 5′-AATCTAGACTATGGACTTGGTGACATGGA-3′; GFP–PigsCHGas2a, NT_PIGSfwd, 5′-CCCCCCCTCGAGATGGCCATGTTAGAGGCGCG-3′ and PigsNTGAS2rv, 5′-GGTAGGGCCCCTAGGAGCTGCGATGTTGGGCACG-3′; GFP–PigsCHGas2b, OE_CG3973_NTfwd, 5′-GCGGCCGCTATGGCCATGTTAGA-3′ and OE_CG3973_NTrev, 5′-ACTAGTTCATGGACTTGGTGACATGG-3′; GFP–PigsCTa, PigsCTonlyfd, 5′-CCCCCCCTCGAGATGGCCCAACATCGCAGCTCCGTGG-3′ and NT_PIGSrev, 5′-GGTAGGGCCCCTAGTAAAGCTCTGTATGATGACG-3′; GFP–PigsCTb, OE_CG3973_CT2fwd, 5′-GCGGCCGCTCGTCGACTAATCGATATG-3′ and OE_CG3973_CT2rev, 5′-ACTAGTTCACTAGTAAAGCTCTGTATGATGAC-3′; GFP–PigsCH-CT, NT_PIGSdCHfwd, 5′-CCCCCCCTCGAGATGGGAGCTGGCTGCTCGGAAAATGGC-3′ and NT_PIGSrev, 5′-GGTAGGGCCCCTAGTAAAGCTCTGTATGATGACG-3′; and GFP–PigsGas2CT, GFP-PigsG2Up, 5′-AAGCGGCCGCCACAATGACGACAATA-3′ and GFP-PigsG2CTLow, 5′-AATCTAGACTAGTAAAGCTCTGTATGATG-3′.

### Cell culture and transfection of *Drosophila* tissue culture cells

S2R+ cells (from the DGRC) were grown in Schneider's medium (Gibco or Lonza) supplemented with 10% heat-inactivated fetal bovine serum (HyClone), 1% penicillin-streptomycin (Sigma), and L-glutamine. Cells were grown at 25°C. S2R+ cells were transiently transfected using Effectene reagent (Qiagen) in 6- or 24-well plates following the manufacturer's instructions. pUASp or pUASTattB plasmids were co-transfected with a constitutive actin5c-Gal4 plasmid in a 1:1 ratio of pUASp:Gal4.

S2 cells (from the DGRC) were maintained and treated with RNAi as described previously (Rogers and Rogers, 2008). Briefly, S2 cells were cultured in SF900II medium supplemented with 100× antibiotic-antimycotic (Life Technologies). RNAi was administered in 6-well plates by treating cells (∼50% confluent) with 10 μg of double-stranded (ds)RNA in 1 ml of medium each day for 7 days.

For all immunofluorescence, S2 and S2R+ cells were plated onto 13-mm glass coverslips coated with concanavalin-A (0.5 mg/ml, Sigma) at 24–48 h after transfection, left to spread for 45 min to 1 h, and then fixed.

### Immunofluorescence

S2R+ cells were fixed for 15 min using 4% formaldehyde in PBS for visualisation of actin, or fixed with either cold methanol for 20 min, or 3% formaldehyde and 90% methanol for 10 min for subsequent MT staining. Cells were then washed three times in PBS, incubated in PBT blocking solution for 20 min [5% bovine serum albumin (BSA, Sigma) and 0.3% Triton X-100 in PBS]. Primary antibodies were added in PBT overnight at 4°C. Coverslips were then washed three times with PBT, and secondary antibodies added for 30 min at room temperature. Fluorescently conjugated phalloidin [Rhodamine–phalloidin (Sigma) or C647-phalloidin (Cambioscience)] was added at 1:500 with secondary antibodies. In some cases, a directly Cy3-conjugated anti-acetylated-tubulin antibody was used. After final washing, coverslips were mounted in Vectashield (Vector Laboratories) or ProLong Gold (Life Technologies). For immunofluorescence of S2 cells, the cells were fixed were fixed 4% paraformaldehyde (EMS, Hatfield, PA) in PEM buffer (100 mM Pipes, EGTA and Mg^2+^) for 15 min at room temperature, followed by three washes in PBS and a blocking step for 15 min in 5% normal goat serum (Sigma-Aldrich) in 0.1% Triton-X 100 (EMS) PBS solution at room temperature for 15 min. Primary antibody, anti-α-tubulin (DM1α, Sigma-Aldrich, diluted to 1:1000 in PBS and 0.1% Triton-X 100), was incubated overnight at 4°C. Secondary antibodies were either incubated overnight at 4°C or for 1 h at room temperature. Secondary antibodies used in this study were conjugated to Alexa Fluor 488 and Alexa Fluor 564 (Jackson ImmunoResearch Laboratories) and used at 1:100 diluted in PBS and 0.1% Triton X-100. Actin was imaged using Alexa-Fluor-488- or Alexa-Fluor-568-conjugated phalloidin (Life Technologies) at 1:100 diluted in PBS and 0.1% Triton X-100. After a final wash in PBS and 0.1% Triton-X-100, the coverslips were mounted with fluorescence mounting solution (Dako, Carpinteria, CA).

Ovaries were fixed for 8 min in 8% formaldehyde in PBS at room temperature for visualisation of actin and MTs, or fixed with 3% formaldehyde and 90% methanol in PBS for 10 min for subsequent MT staining alone.

### Drug treatments

S2R+ cells were plated onto 13-mm glass coverslips coated with concanavalin-A (Con-A, 0.5 mg/ml) at 24–48 h after transfection, left to spread for 45 min, then treated with cytochalasin D (10 μM) in Schneider's medium for 20 min. Cells were fixed using 4% formaldehyde in PBS, washed 2× in PBS, then stained using fluorescently conjugated phalloidin, either Rhodamine–phalloidin (Sigma-Aldrich) or C647–phalloidin (Cambioscience) in PBT.

### Imaging and image processing

For live-cell imaging of S2R+ cells, the cells were replated into four-well glass-bottomed slides (Nunc) coated with Con-A (0.5 mg/ml) and imaged on a Zeiss 780 or Leica SP8 confocal microscope system. Either a *z*-stack was taken with an interval of 0.5 µm, or a time-lapse series of a single plane close to the coverslip was acquired with a time interval of 1–2 s for a total duration of up to 1 min. For live-cell imaging of S2 cells, cells were plated on 0.5 mg/m ConA-treated coverslips attached to drilled 35-mm tissue culture dishes with UV-curable adhesive (Norland Products; Cranbury, NJ) in Schneider's *Drosophila* medium (Life Technologies) supplemented with 10% fetal bovine serum and 100× antibiotic-antimycotic (Life Technologies). Cells were allowed to attach for at least 1 h before imaging. The cells were imaged with a laser TIRF system (Nikon) mounted on an inverted microscope (Ti; Nikon) equipped with a 100×, 1.49 NA objective lens driven by Nikon Elements software. Images were captured with an Andor-Clara Interline camera (Andor Technology, Belfast, UK). All images were processed for brightness and contrast and prepared for publication using Photoshop (CS version 8.0; Adobe Systems).

For live imaging of ovaries, ovaries were dissected from females directly into Voltalef oil on a coverslip. Ovarioles were separated carefully with forceps and then imaged immediately on an inverted Zeiss 780 or Leica SP8 confocal microscope. Either a *z*-stack was taken with an interval of 0.5 µm, or a time-lapse series of a single plane of the squamous follicle cells was acquired with a time interval of 1–2 s for total duration of up to 1 min.

Live confocal imaging was carried out on a Zeiss 780 or Leica SP8 microscope; fixed confocal imaging of both cells and ovaries was carried out on a Zeiss 780 or Olympus Fluoview 1200 microscope, and *z*-stacks of interval 0.5–1 µm were acquired.

Image analysis was carried out in ImageJ, FIJI, Velocity (Perkin Elmer) and Adobe Photoshop.

### *Drosophila* genetics

Transgenic flies were made by injection into embryos of the pUASpGFP-Pigs or pUASTattBGFP-Pigs plasmid DNA constructs (Bestgene, Chino Hills, CA). UAS-lines were crossed to GR1-Gal4 or Cy2-Gal4 for expression in follicle cells, or nanosGal4VP16 for germline expression. To colabel Pigs with CLIP170, stable lines containing pUASpGFP-Pigs and pUASp-mCherry-CLIP170 constructs were generated. The mCherry-CLIP170 line was generated as described in (Stramer et al., 2010). The genomic Pigs–GFP line was generated by deleting all but the first coding exons of Pigs by homologous recombination ends-out targeting. Briefly, homology arms were amplified by PCR from genomic DNA and inserted into the pGX-attP vector (Huang et al., 2009). One 5-kb arm was located upstream of the second coding exon of Pigs, and one 3-kb homology arm was located downstream of the last Pigs exon. Transgenic flies were then generated containing this construct, and homologous recombination induced by crossing with males from line 6934-hid (Huang et al., 2008). Candidate lines were then screened and confirmed by PCR. The Pigs founder line was generated by excising the *w*^+^ (flanked by loxP sites) inserted in the place of the deleted exons, leaving one loxP and an attP site. The genomic region of Pigs containing the deleted exons was amplified and cloned into pGE-attB (Huang et al., 2009), and GFP was inserted at the end of the coding sequence. Finally the PigsGFP^genomic^ line was generated by ΦC31 integration of this construct into the Pigs attP founder line.

### Charge distribution analysis

To analyse the charge distribution in Pigs over small regions, we scanned the amino acid composition of Pigs, giving a value of 1 for each positively charged amino acid, a value of −1 for each negatively charged amino acid and 0 for neutral amino acids. We summed up values over groups of 10 amino acids and assigned the sum to the mid-point of the group, moving the 10-amino-acid window by one residue and repeating the process, to plot all mid-point values.

### Quantification of comet speeds

Comets were analysed using the Manual Tracking plugin in FIJI from time-lapse confocal images of a single *z*-plane for a time interval of 1–2 s. The total distance covered over the course of the tracked comet was divided by the time to give the average speed for each comet. For *in vivo* analysis, 25–56 comets were tracked from 4–5 different ovaries taken from 1–3 independent repeats for each construct. For tissue culture speed analysis, 47–54 comets were tracked from 6 or 7 cells taken from 3–5 independent repeats for each construct.

### Quantification of MT plus-end localisation

Quantitative line scans were performed on individual time frames of movies obtained from live cells coexpressing EB1–RFP and GFP–PigsFL, GFP–PigsSxIP3mut or GFP–PigsSxIP1/2/3mut. Scans and quantifications were performed in ImageJ using the ‘Plot Profile’ function. Fluorescence intensity values for each comet were normalised by dividing the set of intensities by the lowest value. For EB1–RFP and GFP–PigsFL, 16 comets were analysed from seven cells; for EB1–RFP and GFP–PigsSxIP3mut, 9 comets were analysed from three cells; for EB1–RFP and GFP–PigsSxIP1/2/3mut, 14 comets were analysed from four cells.
